# Exploratory MIA study: multimodal magnetic resonance imaging characteristics of rat offspring induced by maternal Poly(I:C) exposure during pregnancy

**DOI:** 10.3389/fpsyt.2025.1710696

**Published:** 2025-12-10

**Authors:** Bin Wu, Yijie Zhang, Yunxia Liu, Zhiwei Feng, Wenjun Sun

**Affiliations:** 1 The Third Clinical Medical College, Beijing University of Chinese Medicine, Beijing, China; 2 Miyun District Hospital of Traditional Chinese Medicine, Beijing, China; 3 Department of Encephalopathy, Beijing University of Chinese Medicine Third Affiliated Hospital, Beijing, China

**Keywords:** arterial spin labeling (ASL), diffusion tensor imaging (DTI), magnetic resonance imaging (MRI), magnetic resonance spectroscopy (MRS), maternal immune activation (MIA), Poly(I:C), schizophrenia

## Abstract

**Background:**

Maternal infection or inflammatory stimulation during pregnancy can disrupt fetal brain development through immune activation, increasing the risk of psychiatric disorders in offspring, including schizophrenia. Using a maternal immune activation (MIA) model induced by polyinosinic–polycytidylic acid (Poly(I:C)) during pregnancy, this study employed multimodal magnetic resonance imaging (MRI)—including T2 structural imaging, diffusion tensor imaging (DTI), arterial spin labeling (ASL), and magnetic resonance spectroscopy (MRS)—to comprehensively assess offspring brain alterations across structure, white matter integrity, cerebral perfusion, and metabolite levels.

**Methods:**

Pregnant Wistar rats were randomly assigned to a Poly(I:C) model group or a saline control group. Adult male offspring underwent T2 structural imaging, DTI, ASL, and MRS. Voxel-based morphometry of T2 images evaluated structural changes; DTI quantified fractional anisotropy (FA) and diffusivity indices (mean diffusivity [MD], axial diffusivity [AD], radial diffusivity [RD]); ASL measured cerebral blood flow (CBF) in the prefrontal cortex and striatum; and MRS assessed metabolite levels in the prefrontal cortex. Group differences were analyzed (*p<*0.05).

**Results:**

Poly(I:C) offspring exhibited significant gray matter density reductions in the prefrontal cortex, hippocampus, striatum, cingulate cortex, and sensorimotor cortex, whereas the right posterior parietal cortex showed increased density and the left third ventricle was enlarged. DTI revealed elevated MD and RD in the hippocampal dentate gyrus, along with increased RD in the genu of the corpus callosum, indicating white matter microstructural damage and abnormalities in myelination. ASL demonstrated significantly increased CBF in the prefrontal cortex and striatum, reflecting abnormal regional perfusion. MRS showed a significant reduction in NAA/Cr in the prefrontal cortex, suggesting impaired neuronal function.

**Conclusion:**

Offspring rats exposed to maternal Poly(I:C) during pregnancy exhibited abnormalities across multiple domains, including brain structure, white matter microstructure, cerebral perfusion, and metabolism. This study provides additional evidence that maternal inflammation during pregnancy can interfere with offspring brain development and impair neurological function.

## 1 Introduction

In recent years, growing evidence from both epidemiological and animal studies has indicated that maternal immune activation (MIA) (1) during pregnancy—triggered by infection or inflammation—may disrupt fetal brain development and substantially increase the risk of schizophrenia (SZ) and other neurodevelopmental disorders in offspring (2). Researchers often use the Polyinosinic-polycytidylic acid (Poly(I:C))-induced MIA model as a neurodevelopmental model for research on processes relevant to schizophrenia (3–5). The offspring of these dams display behavioral alterations relevant to the schizophrenia phenotype, as they relate to underlying cognitive and sensorimotor processes implicated in the disorder, including cognitive impairment (6), social deficits (7), and prepulse inhibition impairments (8). In addition, MIA has also been widely modeled using bacterial lipopolysaccharide (LPS), and the changes it exhibits are similar to those observed when using Poly(I:C) (9–12). The behavioral, neuroanatomical, and neurophysiological alterations observed in these models across rats and mice suggest that the MIA model holds significant value for investigating neurodevelopmental mechanisms relevant to schizophrenia. To further elucidate disease pathophysiology, numerous studies have examined structural alterations in the brains of schizophrenia model rats.

In neuroimaging research, only a limited number of studies have employed magnetic resonance imaging (MRI) to investigate structural brain alterations in Poly(I:C) rats. For example, Piontkewitz et al., using 7T MRI, reported significant volume reductions in several brain regions, including the hippocampus, striatum, and prefrontal cortex, as well as enlargement of the lateral ventricles in Poly(I:C) offspring (13). Similarly, Casquero-Veiga et al. observed volumetric alterations in Poly(I:C)-induced MIA female offspring, including increased gray matter volume in the hippocampus, pituitary gland, and cingulate cortex, reduced gray matter volume in certain thalamic nuclei, cerebellum, and brainstem, alongside enlargement of the lateral ventricles and fourth ventricle (14). Winter et al. (15) utilized ex vivo 7T MRI to demonstrate that adult male Wistar rats with Poly(I:C)-treated offspring had significantly enlarged lateral ventricles, which could be reliably detected by automated segmentation. Romero-Miguel et al. (16) reported ventricular enlargement and hippocampal reduction in the Poly(I:C) model. Liu et al. (17) used 9.4T MRI combined with DTI and MRE to find that Poly(I:C) offspring had enlarged ventricles, abnormally increased white matter diffusion markers (MD, AD, RD), and impaired cortical stiffness development from 4 weeks after birth. Although these studies provide valuable insights, and several research groups have already applied multimodal and whole-brain approaches in the MIA model, many MRI investigations still rely on single imaging modalities or focus on specific brain regions, thereby limiting fully integrative analyses across multiple modalities. This limitation continues to hinder a comprehensive understanding of brain alterations in the Poly(I:C) model.

Diffusion tensor imaging (DTI) is a valuable tool for assessing the microstructural integrity of white matter (18, 19). Previous research has demonstrated that patients with schizophrenia frequently exhibit abnormalities in white matter tracts, including reduced anisotropy (20, 21). In Poly(I:C) rats, Liu et al. reported marked alterations in white matter diffusivity (17). Specifically, they found that with increasing age, Poly(I:C) model rats showed progressive elevations in mean diffusivity (MD) and radial diffusivity (RD) within the corpus callosum, internal capsule, and external capsule, suggesting abnormal myelination or altered cellular density. Di Biase et al. (22) found that male rats exposed to Poly(I:C) prenatally exhibited increased levels of extracellular free water in the corpus callosum and striatum in adulthood, suggesting that extracellular edema and inflammatory responses are involved in white matter changes. Wood et al. (23) did not observe significant differences in DTI during the early developmental stage (P21), suggesting that white matter abnormalities may gradually appear as development progresses. Missault et al. (24), using functional and structural imaging, found that although DTI indices did not change significantly, the default mode network sample region showed excessive synchronization, indicating that functional connectivity abnormalities may precede structural damage. These alterations are consistent with the white matter connectivity deficits commonly reported in schizophrenia, although further longitudinal and multimodal investigations are needed for confirmation.

With respect to functional and perfusion imaging, arterial spin labeling (ASL) MRI provides a noninvasive method for assessing regional cerebral blood flow (25). Although extensive ASL studies have not yet been conducted in the MIA model, early prospective work has suggested that MIA can lead to abnormal cerebral perfusion. For example, an earlier study reported alterations in cerebral perfusion in a Poly(I:C)-treated rat model: Drazanova et al. (26) found that male rats exposed to prenatal Poly(I:C) exhibited hyperperfusion in cerebral circulation regions such as the circle of Willis, hippocampus, and cortical areas. Additionally, Rasile et al. (27) found that Poly(I:C) treatment led to impaired blood-brain barrier function in offspring rats. Thus, ASL offers valuable insights in MIA models for detecting regional alterations in CBF during development or adulthood that may correlate with long-term cognitive, behavioral, or neurometabolic impairments.

From a neurochemical perspective, magnetic resonance spectroscopy (MRS) enables quantification of neurotransmitters and metabolites in the brain (28). Previous studies have reported a significant positive correlation between N-acetylaspartate (NAA) levels in the hippocampus of healthy rats and their performance in hippocampus-dependent spatial memory tasks. Conversely, reduced NAA levels are frequently observed in models of Neurodevelopmental Disorders and Psychiatric Disorders (29). A recent study employing MRS technology revealed that adolescent stress induced a significant decrease in NAA+NAAG levels in the dorsal striatum of MIA (LPS-induced) offspring (30). However, only a few MRS studies have examined the offspring of Poly(I:C)-exposed dams, and it remains unclear whether this model exhibits comparable neurochemical abnormalities.

In summary, while multimodal and whole-brain imaging studies of the MIA model are increasingly reported, many MRI studies of MIA rat models still focus on a single imaging modality or restricted brain regions, and relatively fewer have integrated multiple MRI modalities within the same cohort *in vivo*. To address this gap, the present study employs a multimodal MRI approach to assess brain alterations in the Poly(I:C) model. Using a cohort of Wistar rat offspring prenatally exposed to Poly(I:C), we conducted T2-weighted structural imaging, DTI, ASL, and MRS within the same group, thereby enabling an integrated assessment of changes in brain structure, white matter microstructure, regional perfusion, and neurochemical composition.

## 2 Materials and methods

### 2.1 Animals and drugs

All experimental procedures were conducted in accordance with the *ARRIVE* guidelines and the *Guide for the Care and Use of Laboratory Animals*, and were approved by the *Laboratory Animal Ethics Committee of Beijing University of Chinese Medicine* (Approval Number: BUCM-2024040107-2017). Twelve pregnant female Wistar rats were used in this study. On gestational day 6 (GD6), the animals were purchased from *Beijing Speifu Biotechnology Co., Ltd.* All dams were consistent in gestational age, source, and body weight range. They were housed in the *SPF-grade Laboratory Animal Center of Beijing University of Chinese Medicine* under controlled environmental conditions: a temperature of 22 ± 2 °C, relative humidity of 50–60%, and a 12-h light/dark cycle (lights on at 08:00, off at 20:00). Food and water were provided ad libitum, and the housing environment was maintained in a quiet setting. Throughout the experimental process, efforts were made to minimize stress, and all abnormal behaviors and deaths were carefully recorded.

Some studies have indicated that LPS often leads to premature birth (31–33), while in this study, Poly(I:C) was used as the model agent. Before administration, the dry powder was dissolved in 0.9% sodium chloride injection solution preheated to 50 °C, prepared as a sterile stock solution at a concentration of 5 mg/mL, stored in a protected environment, and thoroughly mixed by vortexing.

### 2.2 Model preparation

#### 2.2.1 Establishment of the MIA model

The Poly(I:C)-induced MIA model was employed as a neurodevelopmental model for studying processes relevant to schizophrenia. After a three-day adjustment period, on gestational GD9 (Critical Periods of Embryonic Development (34)), pregnant rats were randomly assigned to two groups: One group received an intravenous tail vein injection of Poly(I:C) dissolved in 0.9% saline (5 mg/mL) at a dose of 10 mg/kg, whereas the control group received an equivalent volume of saline. The female mice were weighed immediately before injection and again 7 days later. The day of delivery was designated as postnatal day 0 (PD0). Following the method of Fang et al. (35), blood was collected from the tail veins of all female mice approximately 3 hours post-injection. Serum was obtained by centrifuging at 1000×g for 15 minutes at 4 °C. Serum samples were stored at -20 °C until they were analyzed. Serum concentrations of inflammatory cytokines (IL-6, IL-1β, IL-18, and TNF-α) were measured using an ELISA kit (Jiangsu Jingmei) to validate maternal immune activation. Detailed data are available in the **Supplementary Material**.

#### 2.2.2 Grouping

Offspring were weaned on PD21 and housed separately by sex. Male offspring were selected for subsequent experiments. Each cage contained 4–5 rats. To ensure data independence, rats within the same cage were sourced from different litters. Between PD101 and PD110, a series of behavioral tests was performed to evaluate the offspring, thereby confirming the successful establishment of the model. The specific details and procedures for behavioral testing have been published in our previous work (36). We conducted a series of behavioral tests during PD101–110 (proceed in order from low stress to high stress), including the open field test (PD101), Y-maze (PD103), Elevated Plus Maze (PD105), and Pre-pulse Inhibition (PD107-PD108). All behavioral experiments were conducted between 9:00 AM and 6:00 PM, with a one-day interval between each test (Detailed statistics can be found in the **Supplementary Materials**.). After completing all behavioral tests, the rats were transferred to the NMR experimental animal room. Following a 3-day acclimation period, 9 animals were randomly selected from each group—MIA offspring and saline offspring (Control group, CTL)—using a random number table method for MRI testing.

### 2.3 Data acquisition

All MRI experiments were performed on postnatal day 111 (PD111) using a 7.0 T small-animal MRI scanner (Bruker BioSpin, Germany) at the Small Animal *In Vivo* Imaging Laboratory of Capital Medical University. Rats were initially anesthetized in an induction chamber with 5% isoflurane in 95% oxygen, and anesthesia was subsequently maintained at 2% isoflurane in 98% oxygen delivered via a nose cone. Animals were positioned prone on a custom-made animal bed with the head fixed at the isocenter of the radiofrequency coil. Throughout the scanning session, respiration rate was continuously monitored and maintained at 40–60 breaths/min using a small-animal physiological monitoring system (SA Instruments, Stony Brook, NY, USA), and rectal temperature was kept at 36.5–37.5 °C using a warm air heating system.

#### 2.3.1 MRI data acquisition and VBM analysis

High-resolution T2-weighted structural images were acquired using a 2D multi-slice turbo spin-echo sequence with the following parameters: repetition time (TR) = 4200 ms, echo time (TE) = 36 ms, flip angle = 180°, field of view (FOV) = 33 × 33 mm², matrix = 256 × 256, slice thickness = 0.7 mm, no interslice gap, 35 contiguous coronal slices. These images served as an anatomical reference for voxel placement in spectroscopy and region-of-interest (ROI) analyses.

Voxel-based morphometry was conducted on 3D T2-weighted MPRAGE images processed with the spmAnimalIHEP4.1 toolbox (based on SPM12, MATLAB R2014a). Preprocessing steps included (1) spatial registration to a rat brain template using the DARTEL algorithm, (2) tissue segmentation to generate gray matter probability maps, (3) modulation to preserve local volume information, and (4) spatial smoothing with an 8-mm full-width at half-maximum (FWHM) Gaussian kernel. Between-group differences in gray matter density were assessed using voxel-wise two-sample t-tests, with statistical significance set at p *<* 0.05 (cluster-level family-wise error corrected). Significant clusters were reported with MNI coordinates, cluster extent (Ke), and peak T-value. It should be noted that VBM provides measures of relative gray matter density rather than absolute regional volumes.

#### 2.3.2 DTI sequence

Diffusion tensor imaging was performed using a single-shot spin-echo echo-planar imaging sequence in 30 non-collinear directions with the following parameters: TR = 3750 ms, TE = 23 ms, FOV = 35 × 35 mm², matrix = 128 × 128, slice thickness = 1.0 mm, 1.0 mm interslice gap, 12 coronal slices, b-values = 0 and 1000 s/mm², NEX = 1, total scan time is about 17 min 30 s. Automatic higher-order shimming was applied prior to each acquisition. Fractional anisotropy (FA), mean diffusivity (MD), axial diffusivity (AD), and radial diffusivity (RD) maps were generated using ParaVision 5.1 software (Bruker). ROIs were manually delineated on the color-coded FA maps with reference to the Paxinos and Watson rat brain atlas (7th edition) in the following regions: hippocampal subfields (CA1, CA3, dentate gyrus), genu of the corpus callosum, and medial prefrontal cortex. Each ROI was measured three times, and the mean value was used for statistical analysis.

#### 2.3.3 ASL imaging

Cerebral blood flow (CBF) was quantified using the flow-sensitive alternating inversion recovery (FAIR) technique with the following parameters: TR = 18,000 ms, TE = 25 ms, FOV = 33 × 33 mm², matrix = 128 × 128, slice thickness = 2.0 mm, triple inversion recovery time = 22 ms. CBF maps were reconstructed in ParaVision 5.1. ROI-based CBF values were extracted from the prefrontal cortex and striatum using manual segmentation guided by the Paxinos and Watson atlas, with each region measured three times and averaged.

#### 2.3.4 MRS acquisition

Single-voxel ¹H-MRS was performed in the bilateral prefrontal cortex using the point-resolved spectroscopy (PRESS) sequence after acquisition of multiplanar T2-weighted scout images (TR = 4200 ms, TE = 36 ms, matrix = 256 × 256, slice thickness = 0.7 mm, FOV = 33 × 33 mm²). A voxel of 3.0 × 3.0 × 3.0 mm³ was positioned over the bilateral PFC based on anatomical landmarks. Automated shimming and water suppression were performed prior to spectral acquisition. Spectra were acquired with TR = 2500 ms, TE = 136 ms, spectral width = 5000 Hz, and 256 averages. Post-processing was conducted in TopSpin 2.0 software, including Fourier transformation, baseline correction, and peak integration using the residual water signal at 4.7 ppm as chemical shift reference. Metabolite peaks of interest included N-acetylaspartate (NAA, 2.02 ppm), total creatine (Cr, 3.05 ppm), choline-containing compounds (Cho, 3.20 ppm), glutamate (Glu, 3.60–3.80 ppm), and myo-inositol (mI, 3.56 ppm). All metabolite concentrations were expressed as ratios relative to creatine.

### 2.4 Statistical analysis

Experimental data were analyzed using SPSS 23.0 software, and results were expressed as mean ± standard error of the mean. Normality was tested using the Shapiro-Wilk method, and homogeneity of variance was tested using the Levene method. For data conforming to a normal distribution with homogeneous variances, an independent samples t-test was used. For data with unequal variances, a corrected t-test (Welch’s t-test) was applied. Non-normally distributed data were analyzed using the Mann-Whitney U test. In addition, to avoid false positives in multiple experiments, we used the Benjamini–Hochberg (FDR) correction method to adjust the *p* for multiple comparisons. *p<*0.05 was considered statistically significant.

## 3 Results

### 3.1 VBM analysis

**Figure 1:**
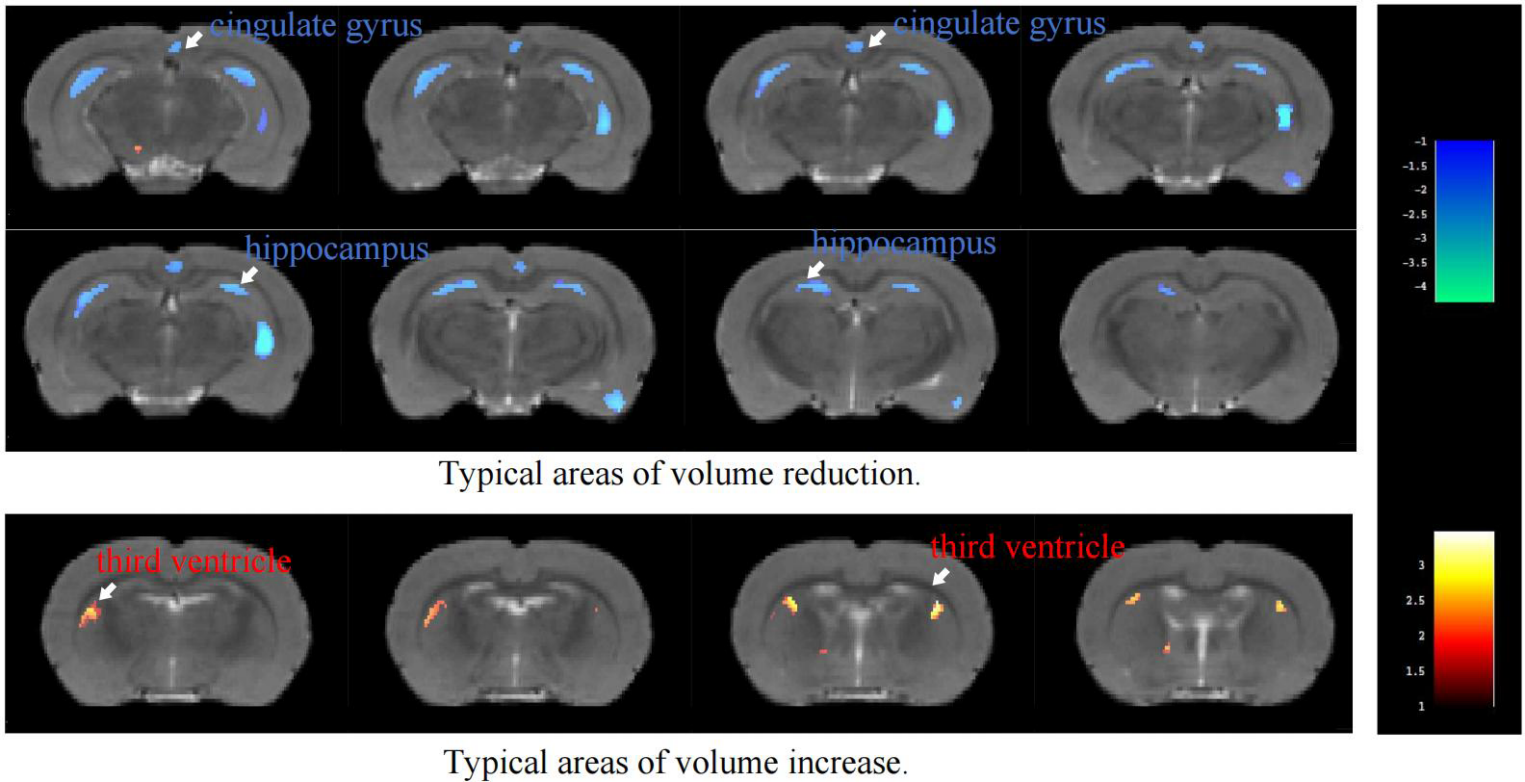
Color-coded VBM overlay images are displayed on the MRI reference map, with warm colors indicating increases and cool colors indicating decreases.

VBM analysis of T2-weighted imaging data revealed significant density alterations in multiple brain regions in the MIA group compared with the CTL group, as illustrated in **Figure 1** and summarized in **Table 1**. Compared with the CTL group, the MIA group showed pronounced density reductions (*p<*0.05) in several regions, including the bilateral striatum, corpus callosum, visual cortex, hippocampus, auditory cortex, cingulate cortex, sensory and motor cortices, as well as both prefrontal cortices. In contrast, among regions with increased density, significant changes were detected only in the right posterior parietal cortex and the left third ventricle.

**Table 1:** VBM analysis of T2-weighted imaging in rats ROIs with significant changes.

Group	Cluster	ROI	Cluster size	MaxT	X	Y	Z
MIA vs CTL (Decrease)	1	Left Striatum	59	3.586	-2.8177	5.9317	-0.3579
		Right Striatum		2.3839	2.0158	9.0338	-10.9179
	2	Right Corpus Callosum	30	2.0989	5.423	6.415	-7.0779
		Left Corpus Callosum		2.232	-2.1364	4.8451	-0.8379
	3	Right Visual Cortex	184	3.685	5.1635	5.515	-7.0779
		Left Visual Cortex		3.8242	-5.5213	4.9396	-7.5579
	4	Right Hippocampus	73	2.8166	5.5548	6.9645	-6.5979
		Left Hippocampus		2.3407	-1.0242	5.1627	-3.7179
	5	Right Auditory Cortex	106	3.5349	7.2823	6.0527	-5.1579
		Left Auditory Cortex		2.2824	-7.1698	5.7592	-4.6779
	6	Left Cingulate Gyrus	63	2.6656	-0.3998	4.6868	-1.3179
		Right Cingulate Gyrus		2.931	-0.123	4.7028	-1.3179
	7	Left Somatosensory Cortex	410	4.5739	-4.6663	3.6151	-1.7979
		Right Somatosensory Cortex		4.0076	3.7456	4.0169	-0.8379
	8	Left Motor Cortex	80	2.6003	-1.0768	4.9651	-9.9579
		Right Motor Cortex		3.5153	1.2223	3.6968	-2.2779
	9	Left Prefrontal Cortex	52	2.4375	-0.1624	6.0595	-8.5179
		Right Prefrontal Cortex		2.3664	-0.024	6.0675	-8.5179
MIA vs CTL (Increase)	1	Right Posterior Parietal Cortex	51	3.6166	4.9921	5.294	-4.6779
	2	Left Third Ventricle	50	2.3844	-2.4132	5.9973	-0.8379

Ke represents the total number of pixels with differences within the cluster, MaxT represents the t-value of the point with the maximum difference, and X, Y, Z are the coordinates of the point with the maximum difference.

### 3.2 DTI analysis

#### 3.2.1 FA statistical results

**Figure 2:**
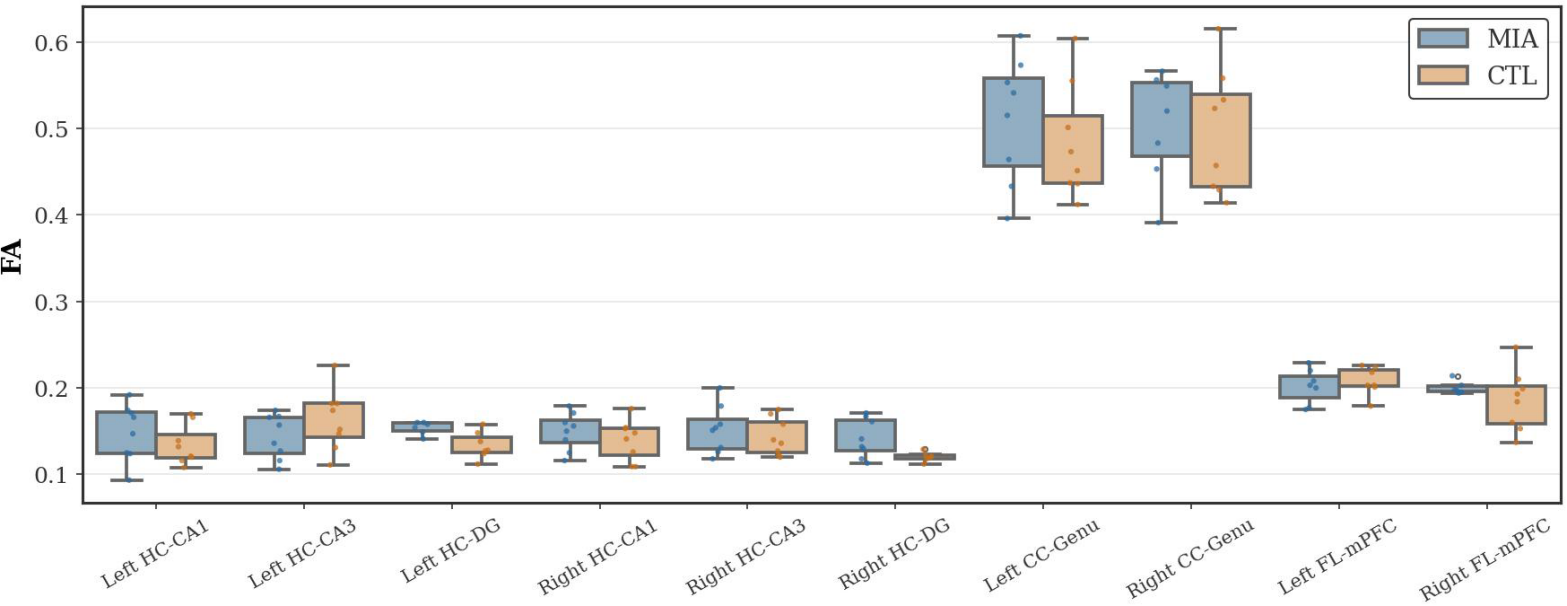
Changes in FA in the hippocampus (HC), corpus callosum (CC), and frontal lobe (FL) of rats in the CTL and MIA groups. “*” indicates p<0.05.

Fractional anisotropy (FA), an indicator of white matter integrity, was analyzed. As **Figure 2** shows, no significant differences in FA values were observed between the groups in the hippocampus, corpus callosum, or frontal lobe.

#### 3.2.2 AD statistical results

**Figure 3:**
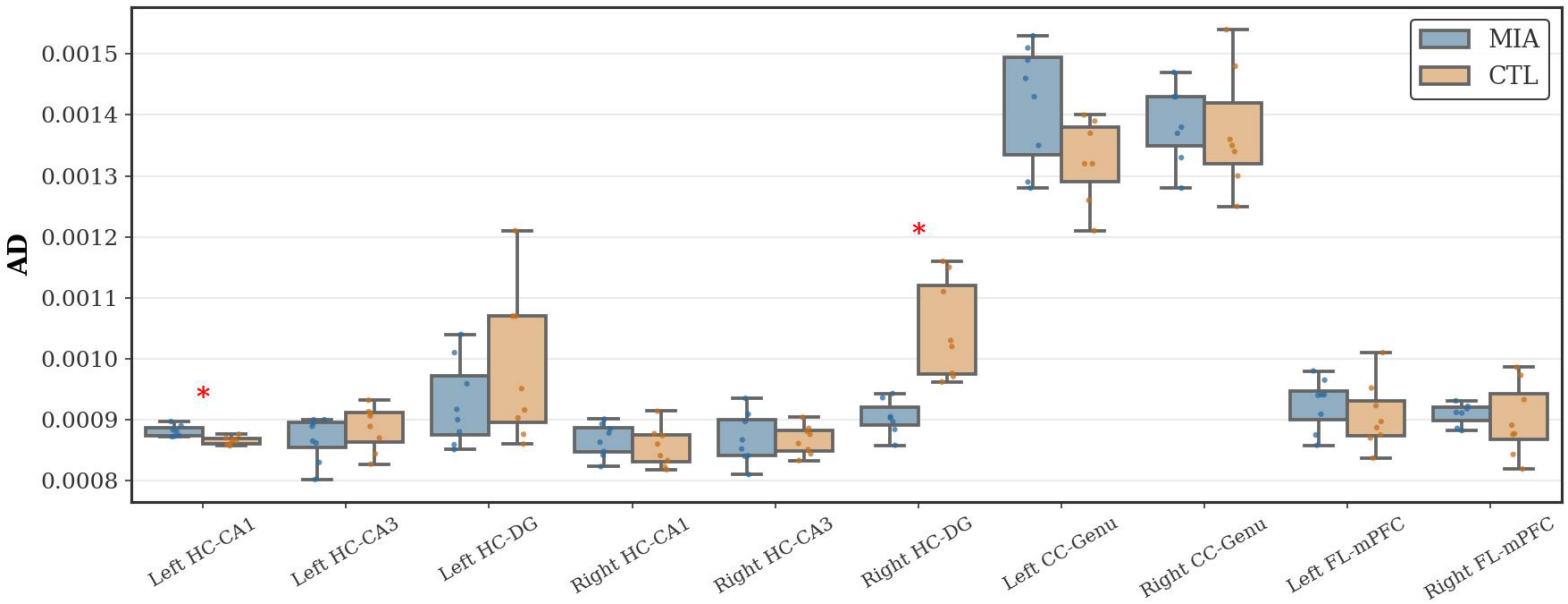
Changes in AD in the hippocampus, corpus callosum, and frontal lobe of rats in the CTL and MIA groups. “*” indicates *p<*0.05.

As **Figure 3** shows, in the hippocampal region, the MIA group showed a significant decrease in AD values in the left CA1 region of the hippocampus (*p<*0.05, Cohen’s d = 2.01, t-statistic = 3.76). In contrast, in the MIA group, the right dentate gyrus exhibited a significant increase in AD values compared with the CTL group (*p<*0.05, Cohen’s d = -2.28, t-statistic = 4.66), indicating altered microstructural integrity. No significant differences in AD values were observed between the groups in the corpus callosum or frontal lobe.

#### 3.2.3 MD statistical results

**Figure 4:**
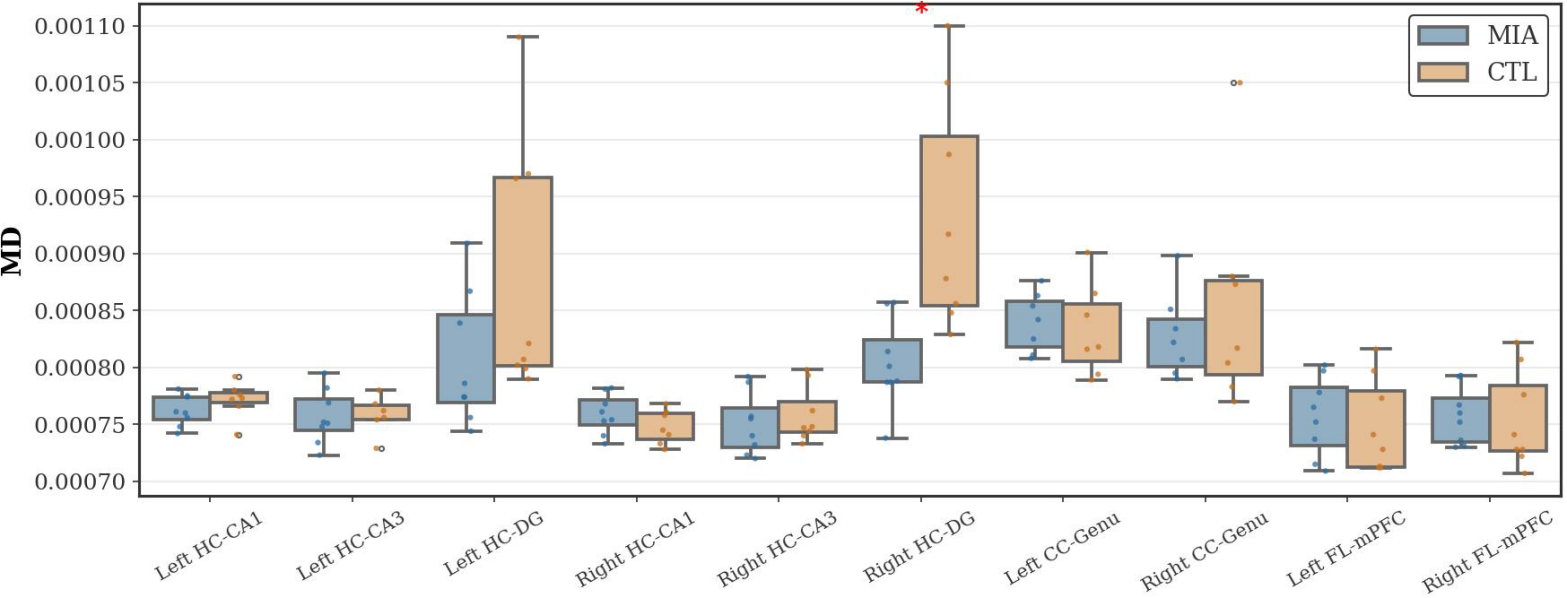
Changes in MD in the hippocampus, corpus callosum, and frontal lobe of rats in the CTL and MIA groups. “*” indicates *p<*0.05.

As **Figure 4** shows, in the hippocampal region, the MIA group showed a significant increase in MD values compared to the CTL group in the right dentate gyrus of the hippocampus (*p<*0.05, Cohen’s d = -1.69, t-statistic = -3.37). No significant differences in MD values were observed between the groups in the frontal lobe or corpus callosum.

#### 3.2.4 RD statistical results

**Figure 5:**
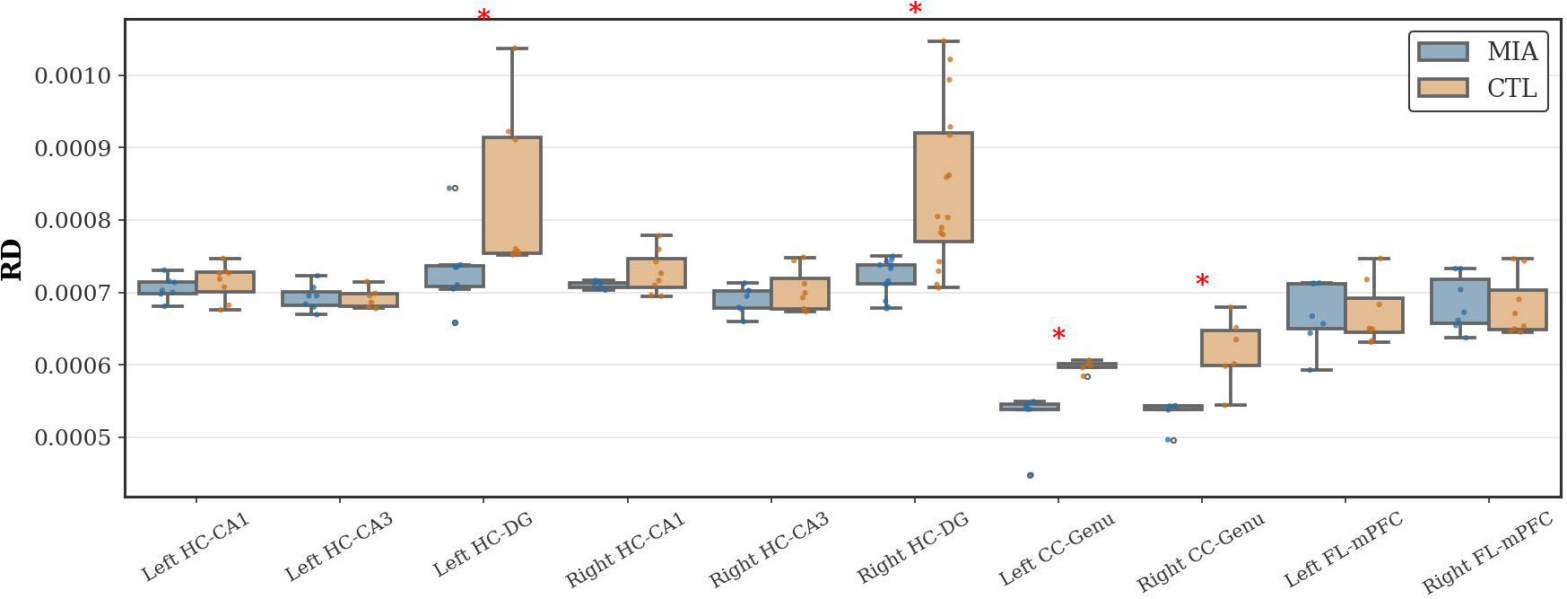
Changes in RD in the hippocampus, corpus callosum, and frontal lobe of rats in the CTL and MIA groups. “*” indicates *p<*0.05.

As **Figure 5** shows, in the hippocampal region, the MIA group exhibited significantly increased RD values compared to the CTL group in both the left and right dentate gyrus of the hippocampus (Left: *p<*0.05, U-statistic = 5.0; Right: *p<*0.05, Cohen’s d = -1.64, t-statistic = -3.39). In the corpus callosum, compared to the CTL group, the MIA group showed significantly increased RD values in both the left and right genu of the corpus callosum (*p<*0.05, U-statistic = 0.0). No significant differences in RD values were observed between the groups in the frontal lobe.

### 3.3 ASL analysis

**Figure 6:**
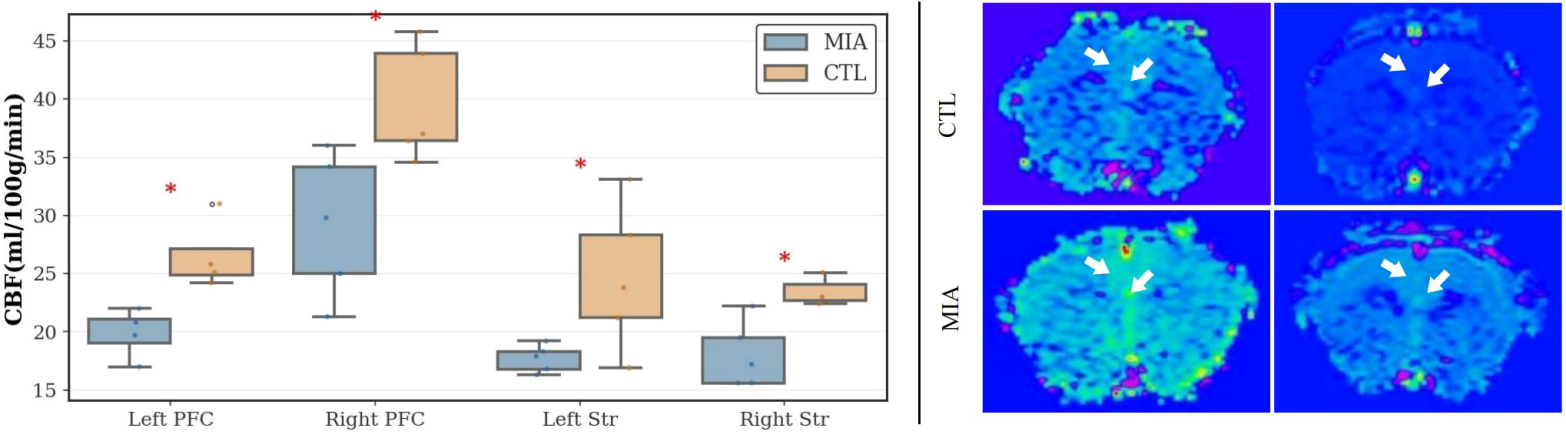
Left: Changes in CBF values in the left and right prefrontal cortex and left and right striatum of the two rat groups. “*” indicates p< 0.05. Right: Representative ASL MR images of rats. The top row represents the CTL group, and the bottom row represents the MIA group. Warm colors (yellow/green/red) indicate higher perfusion, while cool colors (blue) indicate lower perfusion.

As **Figure 6** (left) and **Figure 6** (right), in the prefrontal cortex, compared to the CTL group, the MIA group exhibited significantly increased CBF values in both the left and right frontal lobes (Left: *p<*0.05, Cohen’s d = -2.52, t-statistic = -2.90; Right: *p<*0.05, Cohen’s d = -1.80, t-statistic = -3.57). In the striatum, the MIA group showed significantly increased CBF values relative to the CTL group in both hemispheres (Left: *p<*0.05, Cohen’s d = -1.54, t-statistic = -2.44; Right: *<*0.05, Cohen’s d = -2.24, t-statistic = -3.06).

### 3.4 MRS analysis

**Figure 7:**
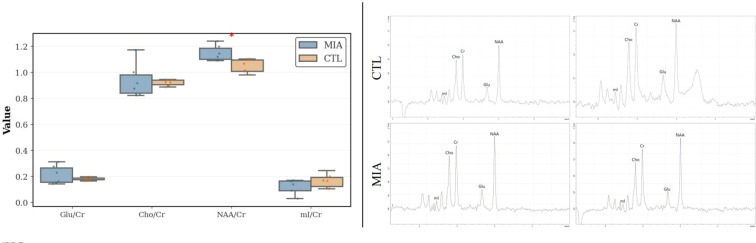
Left: MRS analysis of changes in the two rat groups, showing variations in mI/Cr, Cho/Cr, Glu/Cr, and NAA/Cr ratios. Right: Representative MRS spectra of rats. The top row represents the CTL group, and the bottom row represents the MIA group.

As shown in **Figure 7** (left and right), differential analysis revealed that, compared to the CTL group, the MIA group had a significantly reduced NAA/Cr ratio (*p<*0.05, Cohen’s d = 1.80, t-statistic = 3.11). No significant differences were observed for other ratios (mI/Cr, Cho/Cr, Glu/Cr) or in other inter-group comparisons.

## 4 Discussion

The primary objective of this study was to evaluate imaging alterations in brain regions of the prenatal Poly(I:C) rat model using multimodal MRI. Therefore, in the following discussion, we will address four aspects: T2 structural imaging, DTI white matter microstructure, ASL cerebral perfusion, and MRS neurometabolism. We will focus on comparing the similarities and differences between this study and existing animal model research, analyzing changes in brain regions and their potential causes, highlighting the correspondence among multimodal MRI results, and concluding with a summary of the study’s limitations.

### 4.1 T2 structural imaging

VBM analysis of T2-weighted images revealed that the MIA group exhibited significant reductions in gray matter density in multiple brain regions, including the bilateral striatum, corpus callosum, visual cortex, hippocampus, auditory cortex, cingulate cortex, sensory cortex, motor cortex, and prefrontal cortex. In contrast, increased gray matter density was observed in the right posterior parietal cortex, along with enlargement of the left third ventricle. These findings indicate widespread structural abnormalities in the Poly(I:C) model.

Previous studies using the Poly(I:C)-induced MIA rat model have reported structural alterations in several key brain regions. For example, reductions in hippocampal and striatal density have been associated with schizophrenia-like behavioral phenotypes (37). Piontkewitz et al. (13) observed significant reductions in hippocampal, striatal, and prefrontal volumes in enlargement of the ventricles by ventricular enlargement. Liu et al. (17) similarly noted markedly increased ventricular density, decreased gray matter density, and white matter microstructural disruption in offspring following Poly(I:C) exposure using 9.4T MRI combined with DTI and MRE. Romero-Miguel et al. (16) further validated these findings through MRI and metabolic analysis, both indicating that MIA induces extensive brain morphological alterations similar to those observed in schizophrenia. Haker et al. (37) identified through machine learning analysis that microstructural alterations in the male MIA model were concentrated in the prefrontal and corpus callosum, correlating with cognitive impairments. Casquero-Veiga et al. (14), applying VBM in female Poly(I:C) rats, observed significant gray matter elevation in the cingulate cortex, together with enlargement of the lateral and fourth ventricles. They also reported increased white matter density in the posterior cingulate cortex (within the posterior parietal region), suggesting potential abnormalities in this area. Overall, prior research has focused mainly on the prefrontal cortex, hippocampus, striatum, and ventricles, whereas regions such as the posterior parietal cortex have received relatively little attention.

In the present study, VBM revealed novel structural alterations not consistently highlighted in earlier reports (38, 39), namely gray matter reductions in the cingulate cortex and density increases in the right posterior parietal cortex. The posterior parietal hypertrophy contrasts with the atrophy typically observed in schizophrenia and may reflect compensatory remodeling or aberrant developmental processes. The cingulate cortex, a key region for emotional regulation and cognitive control, may contribute to the emotional and cognitive deficits observed in this model. These discrepancies with previous studies may partly result from methodological differences: (1) Administration of Poly I:C at 10 mg/kg on GD9 (compared to 4 mg/kg on GD15 (13, 16)) may cause more pronounced disruption of early neurogenesis; (2) Use of male offspring only (compared to female offspring in Casquero-Veiga et al. (14)); (3) The absence of prenatal omega-3 supplementation (present in Romero-Miguel et al. (16)); (4) Neurodevelopmental MIA versus pharmacological MK801 models (40).

In summary, this study confirms the presence of widespread structural abnormalities in the Poly(I:C) model and identifies novel alterations in the cingulate and posterior parietal cortices. These findings suggest that the model disrupts the development of emotion–cognition circuits, as well as sensory–motor regions. Importantly, the results highlight both consistencies with previous reports and new region-specific alterations, which may arise from methodological differences, model variations, or age-related factors.

### 4.2 DTI white matter microstructure

In the hippocampus, subregions CA1, CA3, and the dentate gyrus were selected as ROIs, forming the classic trisynaptic circuit (DG → CA3 → CA1) (41), which underlies hippocampus-dependent learning and memory (42). Abnormalities in this circuit are closely associated with memory deficits and information-processing impairments in schizophrenia. In the corpus callosum, the genu was chosen as an ROI because it connects the bilateral prefrontal cortices, with its integrity directly influencing prefrontal function. In the frontal lobe, the medial prefrontal cortex was selected as the ROI; medial prefrontal cortex dysfunction is a hallmark feature of schizophrenia.

DTI provides insight into the microstructural integrity of white matter tracts. Previous studies have shown that prenatal exposure to Poly(I:C) can disrupt myelination in the offspring’s brains, leading to white matter pathology in adulthood. For example, a longitudinal study reported that from 4 weeks of age, Poly(I:C) rats exhibited approximately 40% larger ventricular volumes than controls, alongside increased water diffusivity in major white matter tracts—including the corpus callosum, internal capsule, and external capsule—suggesting reduced myelin integrity. The study also observed elevated MD and RD in these tracts, with corresponding decreases in FA, reflecting the extent of white matter fiber tract damage (17). Some findings have been reported in other MIA studies. Di Biase et al. (22) demonstrated elevated extracellular free water in the corpus callosum and striatum of male offspring, with no significant reduction in myelin fatty acids, indicating that early white matter pathology is primarily characterized by extracellular inflammation/edema rather than inflammation-associated demyelination. Similarly, Singh et al. (43) observed impaired myelination and reduced D2 receptor binding in the corpus callosum following early-life Poly(I:C) exposure, linking microstructural injury to dopaminergic dysfunction. Wood et al. (23) and Missault et al. (24) further suggested that early-stage white matter abnormalities may emerge gradually, with functional network hypersynchrony preceding overt structural damage.

Consistent with these findings, we observed imaging evidence of myelin damage in the hippocampus and corpus callosum. Specifically, the Poly(I:C) group exhibited significantly elevated MD and RD in the hippocampal dentate gyrus, along with increased RD in the genu of the corpus callosum. Casquero-Veiga et al. similarly reported metabolic and morphological changes in female Poly(I:C) rats, supporting the impact of MIA on white matter integrity (14).

However, diffusion characteristics in frontal lobe white matter have shown inconsistent results in the literature (16). While some studies have reported increased FA in the frontal lobe of MIA rats, we observed no significant changes in the mPFC ROI, which may be due to differences in ROI selection, subject sex, or age at scanning.

Overall, our DTI results corroborate those of Liu et al. (17), demonstrating widespread white matter microstructural damage in Poly(I:C) offspring, particularly in hippocampus-related pathways critical for learning and memory and in the genu of the corpus callosum. Differences in white matter damage patterns across studies may stem from multiple factors: (1) variations in ROI selection and analysis methods, (2) differences in imaging time points (adolescence vs. adulthood), and (3) batch or strain differences in the animal model.

In summary, DTI provides new evidence of white matter myelin pathology in the Poly(I:C) model and, together with structural imaging, highlights abnormalities in the hippocampus–prefrontal circuitry.

### 4.3 ASL cerebral perfusion

Core cognitive deficits in schizophrenia are closely linked to hypoactivity in the prefrontal cortex (44). Additionally, the striatum, a central component of the basal ganglia, receives projections from the entire cerebral cortex, particularly the prefrontal cortex (45). Therefore, we focused on measuring CBF in the prefrontal cortex and striatum.

ASL provides a noninvasive method to quantify CBF and assess brain functional states. Clinical studies indicate that individuals with schizophrenia often exhibit weakened functional connectivity within the prefrontal-striatal circuit, alongside increased regional glucose metabolism and blood flow in the striatum (40, 46). Research on cerebral perfusion in animal models of MIA remains limited. Drazanova et al. administered ASL to Poly(I:C)-treated rats and observed enlarged lateral ventricles in male offspring, along with increased perfusion in the circle of Willis, hippocampus, and sensorimotor cortex (26). These findings suggest that MIA may induce hyperperfusion in specific brain regions, potentially reflecting overactivation of dopaminergic or glutamatergic pathways.

In this study, CBF measurements revealed significantly higher perfusion in the bilateral prefrontal cortex and striatum of Poly(I:C) male offspring relative to controls, indicating localized perfusion abnormalities. Our results partially align with those of Drazanova et al., who observed ventricular enlargement and increased perfusion in major brain regions. Similarly, studies using the methylazoxymethanol (MAM) model have reported increased CBF across multiple brain regions, consistent with our findings (26).

Multimodal integration suggests that structural atrophy and elevated blood flow can coexist in the Poly(I:C) model. For instance, the prefrontal cortex showed reduced volume alongside increased local perfusion, suggesting that structurally compromised regions may maintain activity via compensatory hyperactivation. This pattern mirrors the inefficient compensatory hyperperfusion observed in the prefrontal cortex of schizophrenia patients during early disease stages (47), indicating that the MIA model recapitulates this pathological feature.

In summary, the ASL results provide evidence of functional overactivation in the Poly(I:C) model. Combined with structural and DTI findings, these results offer a more comprehensive understanding of brain alterations induced by MIA.

### 4.4 MRS neurometabolites

MRS enables quantitative assessment of brain metabolite levels, reflecting neuronal and glial function. In this study, we examined relative changes in NAA, Cho, Glu, and mI in the prefrontal cortex. The results showed a significant decrease in NAA/Cr in the Poly(I:C) group, whereas no significant differences were observed for Cho/Cr, Glu/Cr, or mI/Cr.

NAA is widely recognized as a biomarker reflecting neuronal integrity and mitochondrial function, with its reduction indicating neuronal injury, dysfunction, or loss (48). This finding is consistent with ¹H-MRS studies of clinical schizophrenia, which frequently report significantly reduced NAA levels in the prefrontal cortex, supporting the pathological hypothesis of abnormal metabolism and function in prefrontal neurons (49). Previous animal studies similarly support metabolic abnormalities in MIA models. For example, Vernon et al. conducted longitudinal MRS tracking in rats and found that gestational Poly(I:C) exposure led to significantly reduced glutathione and taurine levels in the prefrontal cortex of adult offspring rats (50). This finding is consistent with our observations. Recent studies have confirmed that reduced NAA/Cr ratios are among the most reproducible neurochemical signatures of schizophrenia-related pathology (51).

The absence of significant changes in other metabolites does not exclude their involvement in schizophrenia pathophysiology. For instance, Mei et al. (52) reported glial regulation of Glu, which may not have been detected in this study due to the timing of measurements (adulthood) or the limited sample size, potentially missing early inflammation-related changes such as elevated Cho or mI. Similarly, the lack of Glu differences does not invalidate the glutamate hypothesis (53), but may reflect a subclinical or early-stage model.

Overall, the reduction in NAA/Cr provides a robust metabolic marker, reinforcing the relevance of the Poly(I:C) model. Among the metabolites examined, the decline in NAA/Cr represents the most consistent evidence of neuronal compromise, while other metabolites merit further investigation in longitudinal studies with larger cohorts.

### 4.5 Multimodal analysis

Findings across multiple MRI modalities provide mutually supportive evidence of brain alterations in the Poly(I:C) model. In the prefrontal cortex—a hub for cognitive control and one of the regions most vulnerable to maternal immune activation (54)—we observed concurrent gray matter reduction (T2) and decreased NAA levels (MRS). Rather than representing independent abnormalities, these two findings may indicate a shared biological origin, involving impaired neuronal integrity and compromised energy metabolism, likely through disrupted synaptic development, altered glial–neuronal interactions (55), or impaired myelination (56).

Ventricular enlargement detected by structural MRI also fits into this framework. The surrounding white-matter regions exhibited elevated diffusivity on DTI, suggesting axonal or myelin degeneration. This spatial correspondence implies that ventricular expansion may partly result from periventricular tissue loss, reflecting a chronic neurodevelopmental trajectory rather than an isolated structural change (17).

Meanwhile, increased local perfusion, as identified by arterial spin labeling (ASL), provides complementary functional insight, indicating potential compensatory or pathological hyperactivation in structurally compromised regions.

These subtle differences, though minor in degree, representatively embody the characteristics of the MIA model and consistently involve the prefrontal cortex-hippocampus-striatum circuit, thereby facilitating behavioral phenotypes through stable neurobiological transformations (57).

Taken together, the complementary abnormalities across T2, DTI, ASL, and MRS imaging form a coherent picture of MIA-induced pathology: early immune-driven neurodevelopmental disruptions leading to long-term deficits in neuronal viability, myelin integrity, vascular function, and circuit-level activity. This integrated multimodal approach therefore provides a more complete framework for understanding how maternal inflammation shapes the interconnected structural, metabolic, and functional networks relevant to schizophrenia.

### 4.6 Study limitations

First, the limited sample size (nine rats per group) may increase the risk of incidental findings. In our study, our statistical results were based on group-level comparisons and did not indicate the presence of clearly distinguishable subtypes within the groups. Second, the cross-sectional design, with assessment at a single adult time point, precludes evaluation of dynamic developmental trajectories of brain changes. Third, manual segmentation of DTI ROIs, although precise, involves subjectivity that may affect reproducibility. Fourth, this study did not directly measure immune markers, inflammatory cytokines, or neurotransmitter levels, limiting mechanistic validation. Fifth, while four MRI modalities were included, additional approaches—such as functional connectivity—were not assessed, which could provide further insight. Sixth, inherent resolution limitations of MRI may preclude detection of microscopic pathological changes, necessitating complementary histological analyses or higher-field imaging techniques. Seventh, another limitation of this study is the relatively large thickness of the MRI slices, which may lead to a partial volume effect, especially in small structures of the rat brain, potentially affecting the accuracy of the results. Future research could use higher resolution (e.g., 0.5 mm slices) or ultra-high field MRI to mitigate this problem. Finally, a major limitation is that only male offspring were included. Although the decision was initially motivated by the greater stability of PPI deficits in males, this restriction significantly constrains the generalizability of our findings. Prior literature indicates robust sex differences in neurodevelopmental responses to maternal immune activation, suggesting that MIA-induced abnormalities may be sexually dimorphic. Future work should systematically include both male and female offspring to determine whether the multimodal MRI alterations observed here exhibit sex-specific patterns and to clarify how such differences may relate to the known sex disparities in schizophrenia.

Despite these limitations, the present findings offer valuable multimodal evidence of MIA-induced neurodevelopmental pathology and provide a foundation for more comprehensive, sex-inclusive, and mechanistically integrated future investigations.

## 5 Conclusion

This study systematically characterized brain pathological features in a Poly(I:C)-induced maternal immune activation (MIA) model, using multimodal MRI, including structural imaging, DTI, ASL, and MRS. We observed significant volume reductions, white matter microstructural damage, regional hyperperfusion, and neurometabolic abnormalities in key brain regions, highlighting pathological alterations within the prefrontal cortex–hippocampus–striatum circuit. Future studies employing larger and more diverse cohorts, longitudinal designs, molecular and behavioral biomarkers, and inclusion of both sexes will be essential to further elucidate the developmental trajectories, biological mechanisms, and clinical relevance of MIA-induced brain changes. Collectively, the present work contributes to a growing body of evidence positioning the MIA model as a valuable tool for investigating neurodevelopmental mechanisms relevant to schizophrenia and related psychiatric disorders.

## Data Availability

The original contributions presented in the study are included in the article/**Supplementary Material**. Further inquiries can be directed to the corresponding author.
