# Correction: Exploratory MIA study: multimodal magnetic resonance imaging characteristics of rat offspring induced by maternal Poly(I:C) exposure during pregnancy

**DOI:** 10.3389/fpsyt.2026.1782780

**Published:** 2026-01-30

**Authors:** Bin Wu, Yijie Zhang, Yunxia Liu, Zhiwei Feng, Wenjun Sun

**Affiliations:** 1 The Third Clinical Medical College, Beijing University of Chinese Medicine, Beijing, China; 2 Miyun District Hospital of Traditional Chinese Medicine, Beijing, China; 3 Department of Encephalopathy, Beijing University of Chinese Medicine Third Affiliated Hospital, Beijing, China

**Keywords:** arterial spin labeling (ASL), diffusion tensor imaging (DTI), magnetic resonance imaging (MRI), magnetic resonance spectroscopy (MRS), maternal immune activation (MIA), Poly(I:C), schizophrenia

There was a mistake in **Figure 1** as published. **Figure 1** and **Figure 6** were reversed, and there were some minor errors in the caption of **Figure 1**. The corrected **Figure 1** appears below. The correct caption is as follows:

**Figure 1:**
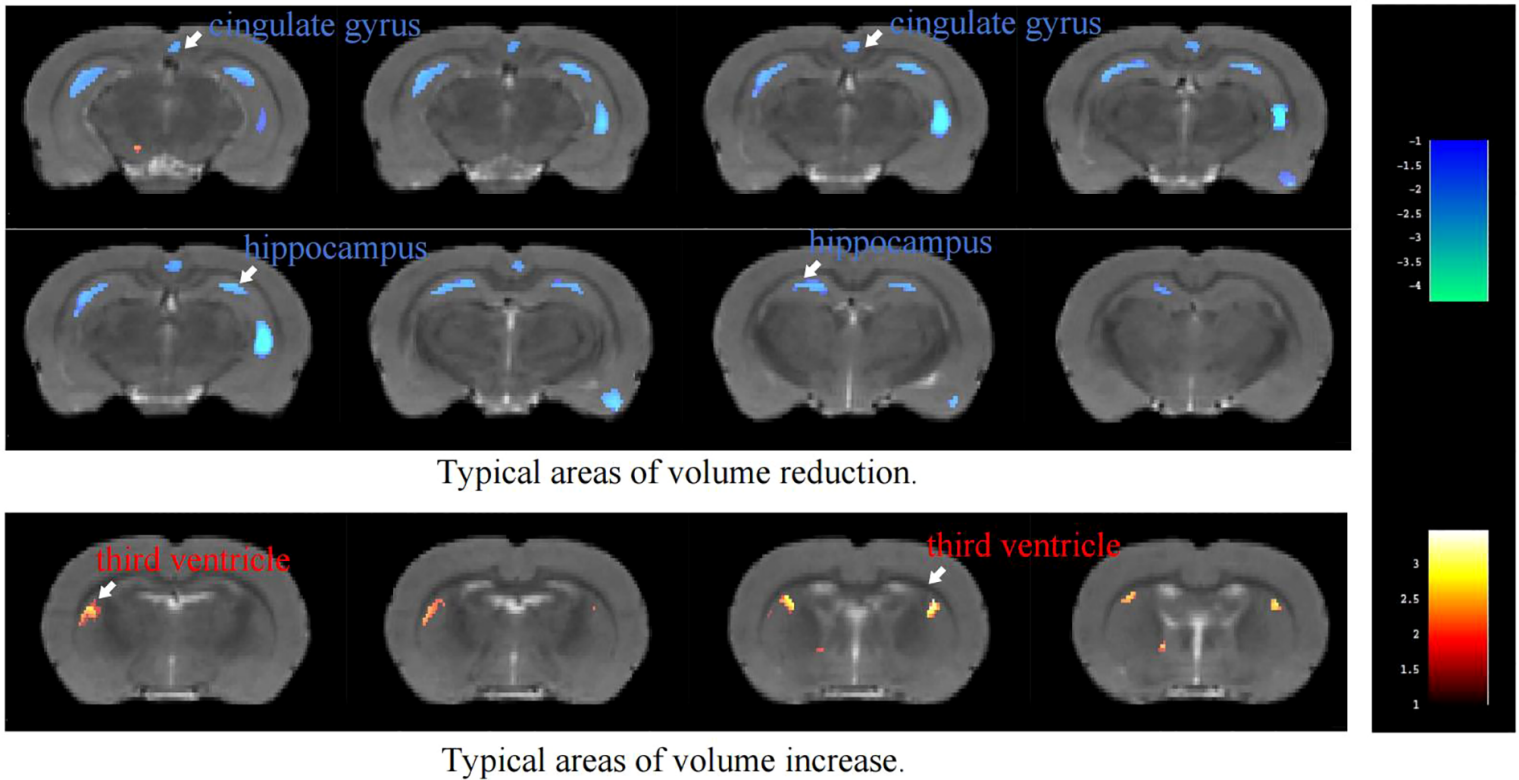
Color-coded VBM overlay images are displayed on the MRI reference map, with warm colors indicating increases and cool colors indicating decreases.

**Figure 6:**
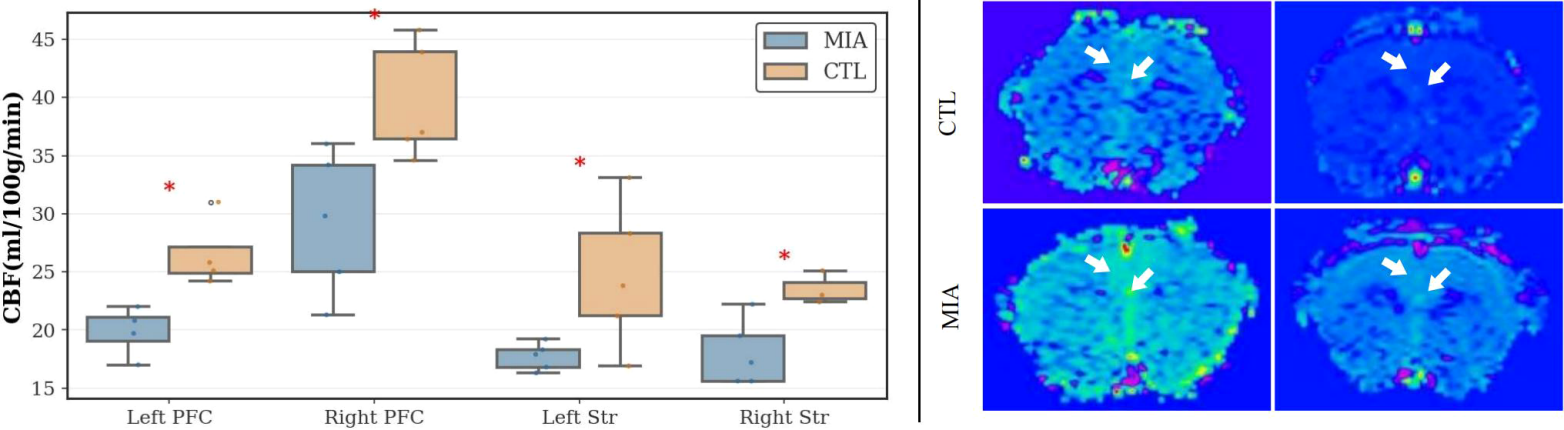
Left: Changes in CBF values in the left and right prefrontal cortex and left and right striatum of the two rat groups. “*” indicates p<0.05. Right: Representative ASL MR images of rats. The top row represents the CTL group, and the bottom row represents the MIA group. Warm colors (yellow/green/red) indicate higher perfusion, while cool colors (blue) indicate lower perfusion.

“**Figure 1**. Color-coded VBM overlay images are displayed on the MRI reference map, with warm colors indicating increases and cool colors indicating decreases.”

Figure 2 and Figure 3 appeared in the wrong order. The order has now been corrected.

There was a mistake in **Figure 6** as published. **Figure 1** and **Figure 6** were reversed, and there are some minor errors in the caption of **Figure 6**. The corrected **Figure 6** appears below. The correct caption is as follows:

“**Figure 6**. Left: Changes in CBF values in the left and right prefrontal cortex and left and right striatum of the two rat groups. “*” indicates p<0.05. Right: Representative ASL MR images of rats. The top row represents the CTL group, and the bottom row represents the MIA group. Warm colors (yellow/green/red) indicate higher perfusion, while cool colors (blue) indicate lower perfusion.”

There was a mistake in **Figure 7** as published. **Figure 7** incorrectly used an image from the Supplementary Materials. The corrected **Figure 7** appears below.

**Figure 7:**
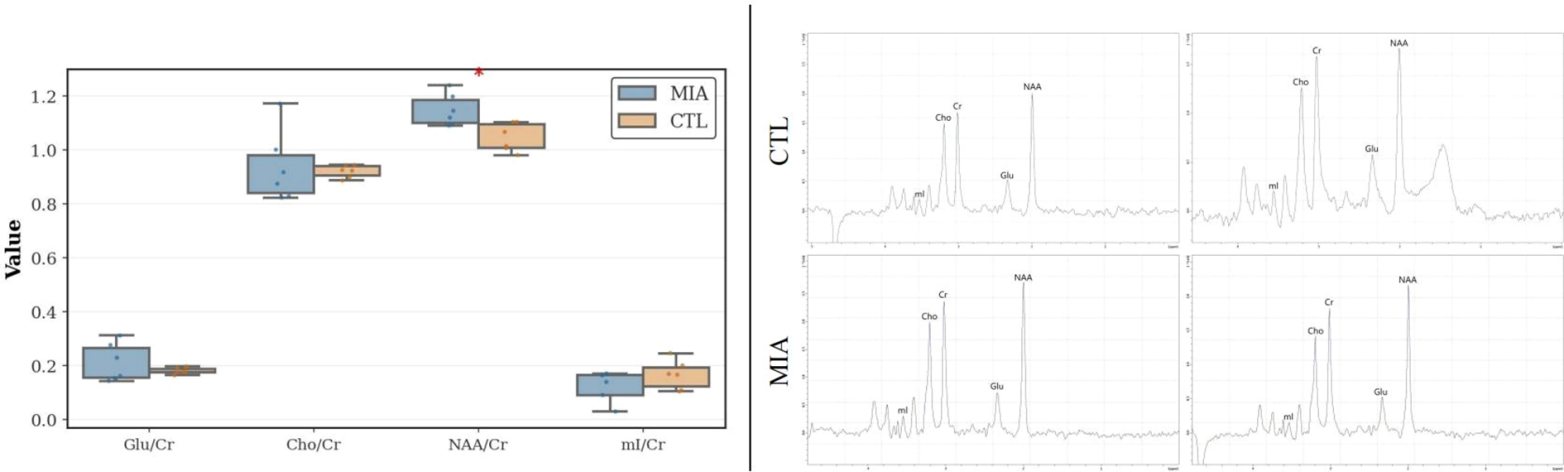
Left: MRS analysis of changes in the two rat groups, showing variations in mI/Cr, Cho/Cr, Glu/Cr, and NAA/Cr ratios. Right: Representative MRS spectra of rats. The top row represents the CTL group, and the bottom row represents the MIA group.

The original version of this article has been updated.

