# Supplementary material for: Exploratory MIA study: multimodal magnetic resonance imaging characteristics of rat offspring induced by maternal Poly(I:C) exposure during pregnancy
Source: Front Psychiatry. 2025 Dec 10;16:1710696. doi: 10.3389/fpsyt.2025.1710696 (PMC12728352; doi:10.3389/fpsyt.2025.1710696)
Supplement: Supplementary file 1 [file DataSheet1.pdf]

Supplementary Material

1 ABBREVIATION LIST

Table S1. Abbreviations and their full definitions used in this study.

Abbreviation	Full definition
MIA	maternal immune activation
Poly(I: C)	polyinosinic–polycytidylic
MRI	magnetic resonance imaging
DTI	diffusion tensor imaging
ASL	arterial spin labeling
MRS	magnetic resonance spectroscopy
FA	ractional1 anisotropy
MD	mean diffusivity
AD	xial diffusivity
RD	radial diffusivity
CBF	cerebral blood flow
SZ	schizophrenia
VBM	Voxel-based morphometry
Ke	cluster size
MaxT	maximum t-value
HC	hippocampus
DG	entate gyrus
CC	orpus callosum
mPFC	medial prefrontal cortex
FL	rontal lobe
PFC	refrontal cortex
Str	striatum
FL	rontal lobe
FL	rontal lobe
ROI	region of interest

2 MRS POSITIONING INSTRUCTIONS

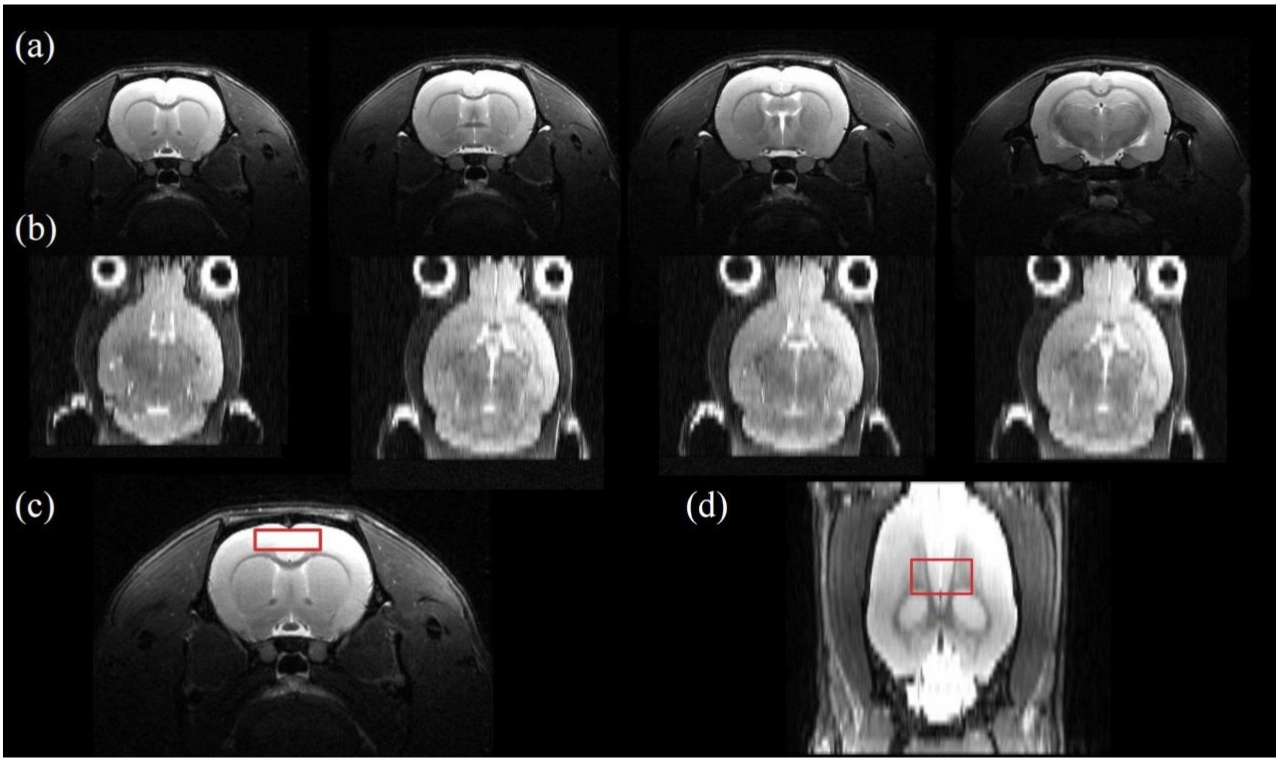

Figure S1. (a-b): Typical multi-slice axial/coronal T2-weighted magnetic resonance imaging. (c-d): The spectral volume of interest (VOI) shown in the red box corresponds to the axial/crown images.

3 STATISTICAL DATA OF MRI

Table S2. Statistical Data of MD

Indicator	Group 1	Group 2	Test Method	t/U	P	FDR-P	Sig(FDR)	Sig	Cohen's d	G1 Mean	G2 Mean	G1 SD	G2 SD
Left HC-CA1	CTL	MIA	Equidistributed t-test	-1.228	0.241	0.603	FALSE	FALSE	-0.635	0.000762	0.000771	1.28e-5	1.45e-5
Left HC-CA3	CTL	MIA	Equidistributed t-test	-0.123	0.905	0.905	FALSE	FALSE	-0.066	0.000757	0.000758	2.25e-5	1.56e-5
Left HC-DG	CTL	MIA	Mann-Whitney U-test	15	0.083	0.414	FALSE	FALSE	—	0.000806	0.000881	5.50e-5	1.06e-4
Right HC-CA1	CTL	MIA	Equidistributed t-test	1.320	0.210	0.603	FALSE	FALSE	0.683	0.000759	0.000748	1.66e-5	1.39e-5
Right HC-CA3	CTL	MIA	Equidistributed t-test	-0.567	0.579	0.877	FALSE	FALSE	-0.284	0.000751	0.000758	2.56e-5	2.29e-5
Right HC-DG	CTL	MIA	Equidistributed t-test	-3.376	0.005	0.045	TRUE	TRUE	-1.688	0.000804	0.000933	3.68e-5	9.47e-5
Left CC-Genu	CTL	MIA	Equidistributed t-test	0.393	0.701	0.877	FALSE	FALSE	0.210	0.000840	0.000833	2.42e-5	3.74e-5
Right CC-Genu	CTL	MIA	Equidistributed t-test	-0.659	0.522	0.877	FALSE	FALSE	-0.352	0.000828	0.000854	3.48e-5	8.90e-5
Left FL-mPFC	CTL	MIA	Equidistributed t-test	0.411	0.687	0.877	FALSE	FALSE	0.206	0.000757	0.000749	3.28e-5	3.86e-5
Right FL-mPFC	CTL	MIA	Equidistributed t-test	0.214	0.834	0.905	FALSE	FALSE	0.107	0.000758	0.000754	2.37e-5	3.98e-5

Table S3. Statistical Data of FA

Indicator	Group 1	Group 2	Test Method	t/U	P	FDR-P	Sig(FDR)	Sig	Cohen's d	G1 Mean	G2 Mean	G1 SD	G2 SD
Left HC-CA1	CTL	MIA	Equidistributed t-test	1.061	0.306	0.613	FALSE	FALSE	0.531	0.149	0.134	0.031	0.022
Left HC-CA3	CTL	MIA	Equidistributed t-test	-1.252	0.231	0.613	FALSE	FALSE	-0.626	0.144	0.163	0.024	0.033
Left HC-DG	CTL	MIA	Equidistributed t-test	2.874	0.015	0.151	FALSE	TRUE	1.599	0.154	0.134	0.007	0.014
Right HC-CA1	CTL	MIA	Equidistributed t-test	0.897	0.385	0.615	FALSE	FALSE	0.448	0.150	0.140	0.020	0.022
Right HC-CA3	CTL	MIA	Equidistributed t-test	0.696	0.498	0.615	FALSE	FALSE	0.348	0.152	0.144	0.026	0.020
Right HC-DG	CTL	MIA	Non-equivariance t-test	2.590	0.032	0.158	FALSE	TRUE	1.221	0.142	0.120	0.021	0.005
Left CC-Genu	CTL	MIA	Equidistributed t-test	0.764	0.458	0.615	FALSE	FALSE	0.382	0.510	0.484	0.068	0.062
Right CC-Genu	CTL	MIA	Equidistributed t-test	0.206	0.840	0.840	FALSE	FALSE	0.107	0.503	0.495	0.059	0.068
Left FL-mPFC	CTL	MIA	Equidistributed t-test	-0.610	0.553	0.615	FALSE	FALSE	-0.326	0.202	0.208	0.019	0.015
Right FL-mPFC	CTL	MIA	Non-equivariance t-test	1.154	0.283	0.613	FALSE	FALSE	0.541	0.200	0.185	0.007	0.033

Table S4. Statistical Data of AD

Indicator	Group 1	Group 2	Test Method	t/U	P	FDR-P	Sig(FDR)	Sig	Cohen's d	G1 Mean	G2 Mean	G1 SD	G2 SD
Left HC-CA1	CTL	MIA	Equidistributed t-test	3.765	0.003	0.013	TRUE	TRUE	2.012	0.000881	0.000865	8.73e-6	6.16e-6
Left HC-CA3	CTL	MIA	Equidistributed t-test	-1.035	0.318	0.637	FALSE	FALSE	-0.517	0.000868	0.000887	3.36e-5	3.42e-5
Left HC-DG	CTL	MIA	Equidistributed t-test	-1.103	0.289	0.637	FALSE	FALSE	-0.551	0.000927	0.000982	6.53e-5	1.15e-4
Right HC-CA1	CTL	MIA	Equidistributed t-test	0.774	0.452	0.646	FALSE	FALSE	0.387	0.000867	0.000855	2.55e-5	3.05e-5
Right HC-CA3	CTL	MIA	Equidistributed t-test	0.111	0.914	0.914	FALSE	FALSE	0.055	0.000869	0.000867	3.90e-5	2.23e-5
Right HC-DG	CTL	MIA	Non-equivariance t-test	-4.660	0.001	0.012	TRUE	TRUE	-2.282	0.000904	0.001047	2.70e-5	7.62e-5
Left CC-Genu	CTL	MIA	Equidistributed t-test	2.079	0.058	0.193	FALSE	FALSE	1.076	0.001418	0.001324	9.23e-5	6.48e-5
Right CC-Genu	CTL	MIA	Equidistributed t-test	0.220	0.830	0.914	FALSE	FALSE	0.117	0.001384	0.001374	6.04e-5	9.38e-5
Left FL-mPFC	CTL	MIA	Equidistributed t-test	0.810	0.431	0.646	FALSE	FALSE	0.405	0.000926	0.000906	3.97e-5	5.08e-5
Right FL-mPFC	CTL	MIA	Non-equivariance t-test	0.411	0.691	0.864	FALSE	FALSE	0.201	0.000909	0.000900	1.69e-5	5.57e-5

Table S5. Statistical Data of RD

Indicator	Group 1	Group 2	Test Method	t/U	P	FDR-P	Sig(FDR)	Sig	Cohen's d	G1 Mean	G2 Mean	G1 SD	G2 SD
Left HC-CA1	CTL	MIA	Equidistributed t-test	-0.763	0.459	0.631	FALSE	FALSE	-0.395	0.000706	0.000714	1.47e-5	2.28e-5
Left HC-CA3	CTL	MIA	Equidistributed t-test	0.130	0.899	0.964	FALSE	FALSE	0.072	0.000694	0.000692	1.65e-5	1.26e-5
Left HC-DG	CTL	MIA	Mann-Whitney U-test	5	0.009	0.028	TRUE	TRUE	—	0.000732	0.000831	5.24e-5	1.04e-4
Right HC-CA1	CTL	MIA	Non-equivariance t-test	-1.623	0.146	0.267	FALSE	FALSE	-0.758	0.000711	0.000728	4.76e-6	2.80e-5
Right HC-CA3	CTL	MIA	Equidistributed t-test	-1.011	0.330	0.519	FALSE	FALSE	-0.523	0.000690	0.000703	1.70e-5	2.77e-5
Right HC-DG	CTL	MIA	Non-equivariance t-test	-3.393	0.010	0.028	TRUE	TRUE	-1.645	0.000724	0.000866	2.22e-5	1.08e-4
Left CC-Genu	CTL	MIA	Mann-Whitney U-test	0	0.008	0.028	TRUE	TRUE	—	0.000528	0.000598	3.55e-5	7.34e-6
Right CC-Genu	CTL	MIA	Mann-Whitney U-test	0	0.008	0.028	TRUE	TRUE	—	0.000533	0.000618	1.82e-5	4.33e-5
Left FL-mPFC	CTL	MIA	Equidistributed t-test	0.046	0.964	0.964	FALSE	FALSE	0.024	0.000671	0.000670	4.18e-5	3.95e-5
Right FL-mPFC	CTL	MIA	Mann-Whitney U-test	31	0.772	0.944	FALSE	FALSE	—	0.000685	0.000681	3.56e-5	3.96e-5

Table S6. Statistical Data

Indicator	Group 1	Group 2	Test Method	t/U	P	FDR-P	Sig(FDR)	Sig	Cohen's d	G1 Mean	G2 Mean	G1 SD	G2 SD
Left PFC	CTL	MIA	Independent Samples t-test	-2.904	0.020	0.030	TRUE	TRUE	-1.837	29.26	39.54	5.51	4.45
Right PFC	CTL	MIA	Independent Samples t-test	-3.569	0.012	0.030	TRUE	TRUE	-2.524	19.88	26.53	1.85	2.65
Left Str	CTL	MIA	Independent Samples t-test	-2.439	0.041	0.041	TRUE	TRUE	-1.543	17.70	24.66	1.04	5.61
Right Str	CTL	MIA	Independent Samples t-test	-3.061	0.022	0.030	TRUE	TRUE	-2.235	18.02	23.50	2.53	1.16

Table S7. Statistical Data of ASL

Indicator	Group 1	Group 2	Test Method	t/U	P	FDR-P	Sig(FDR)	Sig	Cohen's d	G1 Mean	G2 Mean	G1 SD	G2 SD
Left PFC	CTL	MIA	Independent Samples t-test	-2.904	0.020	0.030	TRUE	TRUE	-1.837	29.26	39.54	5.51	4.45
Right PFC	CTL	MIA	Independent Samples t-test	-3.569	0.012	0.030	TRUE	TRUE	-2.524	19.88	26.53	1.85	2.65
Left Str	CTL	MIA	Independent Samples t-test	-2.439	0.041	0.041	TRUE	TRUE	-1.543	17.70	24.66	1.04	5.61
Right Str	CTL	MIA	Independent Samples t-test	-3.061	0.022	0.030	TRUE	TRUE	-2.235	18.02	23.50	2.53	1.16

Table S8. Statistical Data of MRS

Indicator	Group 1	Group 2	Test Method	t/U	P	FDR-P	Sig(FDR)	Sig	Cohen's d	G1 Mean	G2 Mean	G1 SD	G2 SD
Glu	CTL	MIA	Non-equivariance t-test	1.091	0.322	0.430	FALSE	FALSE	0.630	0.212	0.180	0.065	0.011
mI	CTL	MIA	Equidistributed t-test	-1.375	0.202	0.405	FALSE	FALSE	-0.833	0.118	0.165	0.053	0.050
Cho	CTL	MIA	Equidistributed t-test	0.286	0.780	0.780	FALSE	FALSE	0.165	0.936	0.921	0.121	0.021
NAA	CTL	MIA	Equidistributed t-test	3.110	0.011	0.044	TRUE	TRUE	1.795	1.148	1.046	0.055	0.048

4 CYTOKINES

Table S9. Four cytokines in the MIA and CTL groups (Mean ± SD).

Group	IL-1β	IL-18	TNF-α	IL-6
CTL	45.86 ± 3.22	179.72 ± 11.15	393.05 ± 23.36	114.81 ± 9.36
MIA	46.82 ± 2.05	185.70 ± 8.65	404.53 ± 8.98	134.52 ± 6.96
p	0.007***	0.315	0.043**	0.001***
Cohen's d	1.963	0.62	1.827	2.56

Approximately 3 hours after MIA modeling, blood was collected from the tail vein of all female rats. Serum was separated, and IL-6, TNF-α, IL-18, and IL-1β were measured using an ELISA kit. The results showed that inflammatory factors in the MIA group were significantly elevated, demonstrating successful immune activation.

5 BEHAVIOURAL EVALUATION

5.1 Open field test

Locomotor activity and anxiety-like behaviour were assessed using the open field test in a quiet, fully illuminated arena (88 cm × 88 cm × 45 cm). Each rat was placed in the corner facing away from the experimenter and allowed to explore freely for 5 min. Behaviour was recorded and analysed using VisuTrack software (Shanghai Xinruan Information Technology Co., Ltd., China). The following parameters were measured: total distance travelled (cm), average speed (cm/s), time spent in the central zone (30 cm × 30 cm, s), and distance travelled in the central zone (cm). Reduced time and distance in the central area were interpreted as increased anxiety-like behaviour. After each trial, the arena was cleaned with 75% ethanol.

5.2 Y-maze spontaneous alternation test

Spatial working memory and novelty preference were evaluated using a continuous Y-maze spontaneous alternation paradigm. The Y-maze consisted of three identical black arms (50 cm length × 10 cm width × 30 cm height) positioned at 120° to each other. Each rat was placed at the end of a fixed start arm and allowed to explore all three arms freely for 5 min without any prior training or blocked arm. Behaviour was video-recorded and analysed automatically. The percentage of total time spent in the central triangular zone was used as the primary index of spatial working memory and novelty-seeking behaviour. The apparatus was wiped with 75% ethanol between animals.

5.3 Elevated plus maze test

Anxiety-like behaviour was further assessed using the elevated plus maze, which was raised 50 cm above the floor and consisted of two open arms (60 cm × 8 cm), two closed arms (60 cm × 8 cm × 30 cm), and a central platform (8 cm × 8 cm). Each rat was placed in the central zone facing a closed arm and allowed to explore freely for 5 min. An arm entry was defined as 60% of the rat's body entering the arm. The following parameters were calculated as proportions of total exploration: distance travelled in open arms, time spent in open arms, and number of open-arm entries. After each test, the maze was thoroughly cleaned with 75% ethanol.

5.4 Prepulse inhibition

We used the paradigm of prepulse inhibition (PPI) to assess the rats' sensorimotor gating ability. PPI was performed using a single sound-attenuated chamber startle apparatus, and the results were analysed using dedicated software (Shanghai Xinruan Information Technology Co., Ltd., China). One day before testing, all rats were habituated to the chamber with 68 dB background white noise for 5 minutes. The formal session began with a 5-minute acclimatisation period (68 dB background). This was followed by six initial startle pulses (120 dB, 40 ms) to establish a stable baseline. Subsequently, trials of four types were presented pseudo-randomly (10 trials each, average inter-trial interval 15 s): (i) pulse alone (120 dB, 40 ms), (ii) three prepulse intensities (76, 80, or 84 dB, 20 ms) followed 100 ms later by the 120 dB pulse, (iii) prepulse alone, and (iv) no stimulus. The percentage PPI was calculated as [1 − (mean startle amplitude on prepulse+pulse trials / mean startle amplitude on pulse-alone trials)] × 100%.

5.5 Statistical Analysis

The behavioural phenotyping confirmed that prenatal Poly(I:C) administration successfully induced a robust MIA-related neurodevelopmental phenotype in adult offspring (Table S10).

Table S10. Behavioral performance in CTL and MIA groups (Mean ± SD)

Measure	CTL	MIA	p	Cohen's d
**Open Field Test (OF)**				
Total distance (mm)	24177.144 ± 5101.857	18993.748 ± 3915.895	0.032*	1.150
Average speed (mm/s)	143.696 ± 123.467	66.184 ± 15.292	-	0.854
Center time (s)	3.469 ± 2.793	0.741 ± 2.223	0.036*	1.081
Center distance (mm)	454.387 ± 359.693	68.232 ± 204.697	0.017*	1.307
**Y-maze**				
Percentage of time in the novel arm (%)	0.460 ± 0.105	0.248 ± 0.086	0.000***	2.205
**Elevated Plus Maze (EPM)**				
Percentage of open arm distance (%)	0.136 ± 0.081	0.110 ± 0.067	-	0.353
Percentage of open arm duration time (%)	0.105 ± 0.09	0.098 ± 0.073	-	0.088
Percentage of open arm frequencies (%)	0.135 ± 0.041	0.197 ± 0.068	0.041*	1.087
Total distance (%)	19788.628 ± 9130.587	19825.373 ± 7152.834	-	0.004
**Pre-pulse Inhibition (PPI)**				
PPI 76 dB	0.618 ± 0.162	0.454 ± 0.114	0.035*	1.170
PPI 80 dB	0.672 ± 0.158	0.540 ± 0.103	-	0.983
PPI 84 dB	0.670 ± 0.124	0.585 ± 0.096	-	0.769

The open field test (OF) was employed to evaluate spontaneous locomotor activity, exploratory behavior, and anxiety-like behavior in rats. Compared with the control (CTL) group, MIA rats displayed a significant reduction in total distance traveled (CTL: 24177.144 ± 5101.857 mm vs. MIA: 18993.748 ± 3915.895 mm; $p = 0.032$, Cohen's $d = 1.150$), indicating markedly attenuated spontaneous exploratory activity in the MIA group. No significant difference was observed in average movement speed between the two groups ($p > 0.05$). With respect to anxiety-related parameters, MIA rats spent significantly less time in the central zone (CTL: 3.469 ± 2.793 s vs. MIA: 0.741 ± 2.223 s; $p = 0.036$, Cohen's $d = 1.081$) and traveled a significantly shorter distance in the central zone (CTL: 454.387 ± 359.693 mm vs. MIA: 68.232 ± 204.697 mm; $p = 0.017$, Cohen's $d = 1.307$). These results demonstrate pronounced central zone avoidance in MIA rats, indicating a significantly elevated level of anxiety-like behavior.

The Y-maze task is primarily used to evaluate spatial working memory in rats, with the percentage of time spent exploring the novel arm serving as a key indicator of spatial recognition memory. Results showed that MIA rats exhibited a significantly lower percentage of exploration time in the novel arm compared to the CTL group (CTL: 0.460 ± 0.105 vs. MIA: 0.248 ± 0.086, $p < 0.001$, Cohen's $d = 2.205$). These findings indicate that MIA rats displayed impaired preference for exploring the novel environment, reflecting a pronounced deficit in spatial working memory.

Anxiety-like behavior was assessed using the elevated plus maze (EPM). Results showed that the percentage of entries into the open arms was slightly but significantly higher in the MIA group compared to the CTL group (CTL: 0.135 ± 0.041 vs. MIA: 0.197 ± 0.068, $p = 0.041$, Cohen's $d = 1.087$), suggesting mildly reduced anxiety-like behavior on this specific measure. However, there were no significant group differences in the percentage of time spent in the open arms (CTL: 0.105 ± 0.090 vs. MIA: 0.098 ± 0.073, $p > 0.05$) or the percentage of distance traveled in the open arms (CTL: 0.136 ± 0.081 vs. MIA: 0.110 ± 0.067, $p > 0.05$). Additionally, total distance traveled in the EPM did not differ significantly between the two groups (CTL: 19788.628 ± 9130.587 mm vs. MIA: 19825.373 ± 7152.834 mm, $p > 0.05$).

Pre-pulse inhibition (PPI) is a core measure of sensorimotor gating function. Results revealed that at a 76 dB pre-pulse intensity, the PPI percentage was significantly lower in the MIA group compared to the CTL group (CTL: 0.618 ± 0.162 vs. MIA: 0.454 ± 0.114, $p = 0.035$, Cohen's $d = 1.170$). At 80 dB, the MIA group showed a strong trend toward reduced PPI, though it did not reach statistical significance (CTL: 0.672 ± 0.158 vs. MIA: 0.540 ± 0.103). At 84 dB, the difference between groups further diminished ($p > 0.05$). These findings indicate that MIA rats exhibit impaired sensorimotor gating, reflecting a deficit in sensory filtering.

Overall, MIA rats exhibited moderate-to-large effect size differences across multiple core behavioral indices, fully meeting the pre-established criteria (The MIA model was deemed successfully established for each batch only if the model animals displayed significant behavioral abnormalities ($p < 0.05$) in at least two of the above-mentioned behavioral domains.) for successful model establishment.

5.6 Animal numbers

A total of $n = 12$ rats per group were initially included for behavioral phenotyping validation (CTL: $n = 12$; MIA: $n = 12$, model successfully established). To optimize experimental costs, 9 animals from each group were randomly selected for subsequent MRI experiments.
